# A community based intervention to modify preventive behaviors of cutaneous leishmaniasis in children: a randomized controlled trial based on PRECEDE PROCEED model

**DOI:** 10.1186/s12889-024-18810-5

**Published:** 2024-05-13

**Authors:** Hossein Jajarmi, Seyedeh Belin Tavakoli sani, Asma pourtaheri, Mahdi Gholian-Aval, Habibollah Esmaily, Seyed Hamid Hosseini, Rezvan Rajabzadeh, Hadi Tehrani

**Affiliations:** 1 Student Research Committee, Mashhad University of Medical Sciences, Mashhad, Iran; 2 Department of Health, Safety, and environment, School of Health, Mashhad University of Medical Sciences, Mashhad, Iran; 3 Department of Health Education and Health Promotion, School of Health, Mashhad University of Medical Sciences, Mashhad, Iran; 4 Instructor of Health Education, School of Public Health, Bam University of Medical Sciences, Bam, Iran; 5 Department of Biostatistics, Faculty of Health, Mashhad University of Medical Sciences, Mashhad, Iran; 6 Health Education and Promotion, Vector-borne Diseases Research Center, North Khorasan University of Medical Sciences, Bojnurd, Iran; 7 Epidemiology, Vector-borne Diseases Research Center, North Khorasan University of Medical Sciences, Bojnurd, Iran; 8 Social Determinants of Health research center, Mashhad University of Medical Sciences, Mashhad, Iran

**Keywords:** Prevention behavior, Health education, Leishmaniasis, PRECEDE-PROCEED model, Children

## Abstract

**Objectives:**

Iran ranks among the top six countries globally with a significant incidence of Cutaneous Leishmaniasis (CL). Using planning models is one community-based intervention to promote preventive behaviors. The purpose of our study was to evaluate the effectiveness of the PRECEDE-PROCEED model (PPM) in modifying preventive behaviors related to CL in children through mother training in a community intervention.

**Methods:**

A randomized controlled trial based on the PPM model was conducted on 168 mothers (intervention (*n* = 84) and control group (*n* = 84) with 10 years old children in the rural areas of Iran. Mothers from 7 village areas were randomly allocated to the intervention (2 village) and control groups (5 village). The intervention group received a program comprising eight 90-minute training sessions and environmental interventions. In this study, we utilized the PPM as a framework to design the questionnaires on Leishmaniosis prevention behavior. Participants in both groups completed the questionnaires at baseline (before the intervention), immediately after the intervention, and at the 2-month follow-up. Analysis of the data was conducted utilizing SPSS_20_, with statistical significance set at *p* < 0.05.

**Results:**

Compared to the control group, the intervention group showed significant increases in knowledge, enabling factors, reinforcing factors, attitude, and preventive behaviors related to Cutaneous Leishmaniasis over time from baseline to follow-up (*P* < 0.001). No significant differences (*P* > 0.05) were observed in the alterations of the PPM construct, knowledge, and preventive behaviors within the control group from pre-intervention to follow-up.

**Conclusions:**

Community (education and environmental) intervention based on PPM is feasible and acceptable to modify preventive behaviors of Cutaneous Leishmaniasis in children by increasing a mother’s knowledge and attitude as well as changing enabling and reinforcing factors.

**Trial registration:**

IRCT20160619028529N8.

## Introduction

Leishmaniasis is a worldwide vector-borne diseases caused by parasites belonging to the trypanosome genus Leishmania [1, 2]. Globally, around 12 million individuals are afflicted with leishmaniosis, with 1.5-2 million new cases emerging annually [3]. In 2020, more than 85% of new cases were reported in ten countries: Afghanistan, Pakistan, Algeria, Iran, Iraq, Brazil, Colombia, Libya, Syria, Tanzani [2, 4].

CL imposes a substantial health burden due to prolonged wound duration, formation of unwanted scars, risk of secondary infections, high treatment costs for communities, lengthy treatment courses, and treatment-related side effects [5]. Likewise, there are no specific guidelines for CL treatment in the pediatric population. Advanced interventions are typically employed for the treatment and testing of adults with CL [6].

Iran ranks among the top six countries globally with a significant incidence of CL. Based on information from the Ministry of Health of Iran, the annual incidence of CL is estimated to range from 50 to 250 cases per 100,000 population in various rural and urban areas of the country [2, 7]. From an epidemiological perspective, the true incidence of CL is likely higher due to underreporting in the rural areas of the country. The Northern Khorasan province in Iran is an endemic area for leishmaniosis. At least 2831 cases of cutaneous leishmaniosis were reported in different regions of the province, with children aged 0–15 accounting for 41.3% of the total individuals infected with CL [2, 8]. Animal husbandry being the primary occupation and the presence of various rodent species are linked to higher CL rates in this region. Other challenge caused severs prevalence of CL in study area related to COVID-19 pandemic in December 2019 [9, 10].

Several studies reported that specific intervention has a significant effect in promote health behaviors and main solutions to address social determinants of health that influence control and prevention of disease [11, 12]. Therefore, the subject of health education is one of the most important principles of the WHO control plan. Although the model has been shown to be effective in promoting preventative behavior but has received less attention in promoting leishmaniosis prevention behaviors [13, 14]. Training children to perform preventative behaviors comes with many challenges. Mothers’ education can help to promote preventative behavior and reduce the burden of disease in children [15]. The close relationship between mother and child makes them known as one of the most important caregivers of the child [16, 17]. Therefore, mothers can play a vital role in preventing, managing and controlling the CL [18, 19].

There is growing global evidence reporting that theory-based intervention is practical solution to modify disease prevention behaviors and complications [20–22]. The effectiveness of health educational intervention depends on the correct selection of theories or models and their application in the health intervention [10, 23]. The PRECEDE-PROCEED Model (PPM) is a comprehensive framework utilized to evaluate health needs, enabling the design, implementation, and assessment of health promotion and other public health programs tailored to address these needs [24, 25]. . The results of more studies show that the application of PPM is successful to prevent and control infection disease because a person’s motivation and decision to adopt a healthy behavior depends on moderating behaviors [11, 21], personal perception [10, 11], and the likelihood of doing that behavior [11, 26].

Although in Iran, several health program and preventive measures have been implemented to controlling CL since 1977, the burden and annual incidence of CL are still high in different parts of the Iran. Likewise, controlling CL is much more difficult in endemic areas because of the complexity of the biological and epidemiological characteristics of CL. It was evidenced that inappropriate environment and low individual’s knowledge and attitude about preventive behaviors of CL are the main barrier to control and prevent CL in Iran [27, 28]. Despite the efficacy of the health educational training to modify disease prevention behaviors, little interventional study has linked mother’s training with preventive behaviors of CL in children. Children are the primary at-risk group for infectious diseases like CL due to their unique behaviors and biological characteristics [29]. Children are more exposed to sandflies, and it is also difficult to diagnose the disease and this delays the healing and spread of wounds [30]. Also, children do not have good cooperation for treatment and usually their treatment period is not completed [5]. Although, children face serious risk related to CL, little is understood about how, where, why, and when children are exposed to CL. It is essential to conduct a series of pilot-scale and modeling researches to better understanding social and environmental sources and other factors that that influence most children’s exposures to CL [11, 17]. It is still unclear how/what theory-based educational intervention can promote individual’s preventive behaviors and skills to control and prevent burden and actual incidence of CL [17, 18]. Thus, it is essential for health manager and policy maker to take step to assess training strategies, which improve individual’s preventive behaviors toward CL and health outcomes in the medical care context. To address this gap, this study aims to evaluate the effectiveness of a community intervention based on the PPM on modifying the preventive behaviors of CL in children through mother education. In this study, we considered the effectiveness of the educational and environmental intervention on behavior prevention of leishmaniosis as the primary outcome. The secondary outcome of this study was to investigate the effect of intervention on structure of PPM.

## Methods

### Study design

This is a randomized controlled trial to evaluate the effectiveness of a community intervention based on PPM to leishmaniosis prevention behaviors in north Khorasan Iran. consists of two main components: PRECEDE and PROCEED. PRECEDE stands for Predisposing, Reinforcing, and Enabling Constructs in Educational Diagnosis and Evaluation, and it consists of four phases: Social diagnosis, Epidemiological diagnosis, Behavioral and environmental diagnosis, educational diagnosis. PROCEED stands for Policy, Regulatory, and Organizational Constructs in Educational and Environmental Development, and it consists of four phases: Implementation, Process evaluation, Impact evaluation, Outcome evaluation.

In this study, as the health issue (leishmaniasis) was already well-defined, the initial social and epidemiological diagnosis stages were deemed unnecessary. Additionally, due to the extended follow-up period, the outcome evaluation phase did not require the final stage of the model. Therefore, the study was executed using the remaining five phases of the PRECEDE-PROCEED model.

### Participant and sampling

Children do not cooperate well in educational interventions Therefore, mothers who have children under the age of 10, who are called parent, are used as the research population [31]. The sampling process was conducted using cluster sampling. so that out of 13 villages, 7 villages randomly were included in the study in clusters. There were 2 villages in the intervention group (*n* = 84) and 5 villages in the control group(*n* = 84).

In this study mothers were included if they: (a) had a child under 10 years old, (b) did not have leishmaniosis in children, (c) were residence for at least 6 months in the area, and (d) were able to complete the questionnaire. They were excluded if mother (a) unwilling to attend, (b) absent from more than one training session, and (c) had suffered mental illness. To estimate the sample size for this study, the mean and standard deviation change in behavior scores between two groups from a similar study by Omoidi will be used as a reference [32]. Thus, we calculated the sample size to be 70 participants in each group with consideration α = 0·05, $\beta$=0.2, Finally, Taking into account the 20% drop rate, the final sample size of 84 people in each group was estimated,

### Randomization and recruitment

**Fig. 1:**
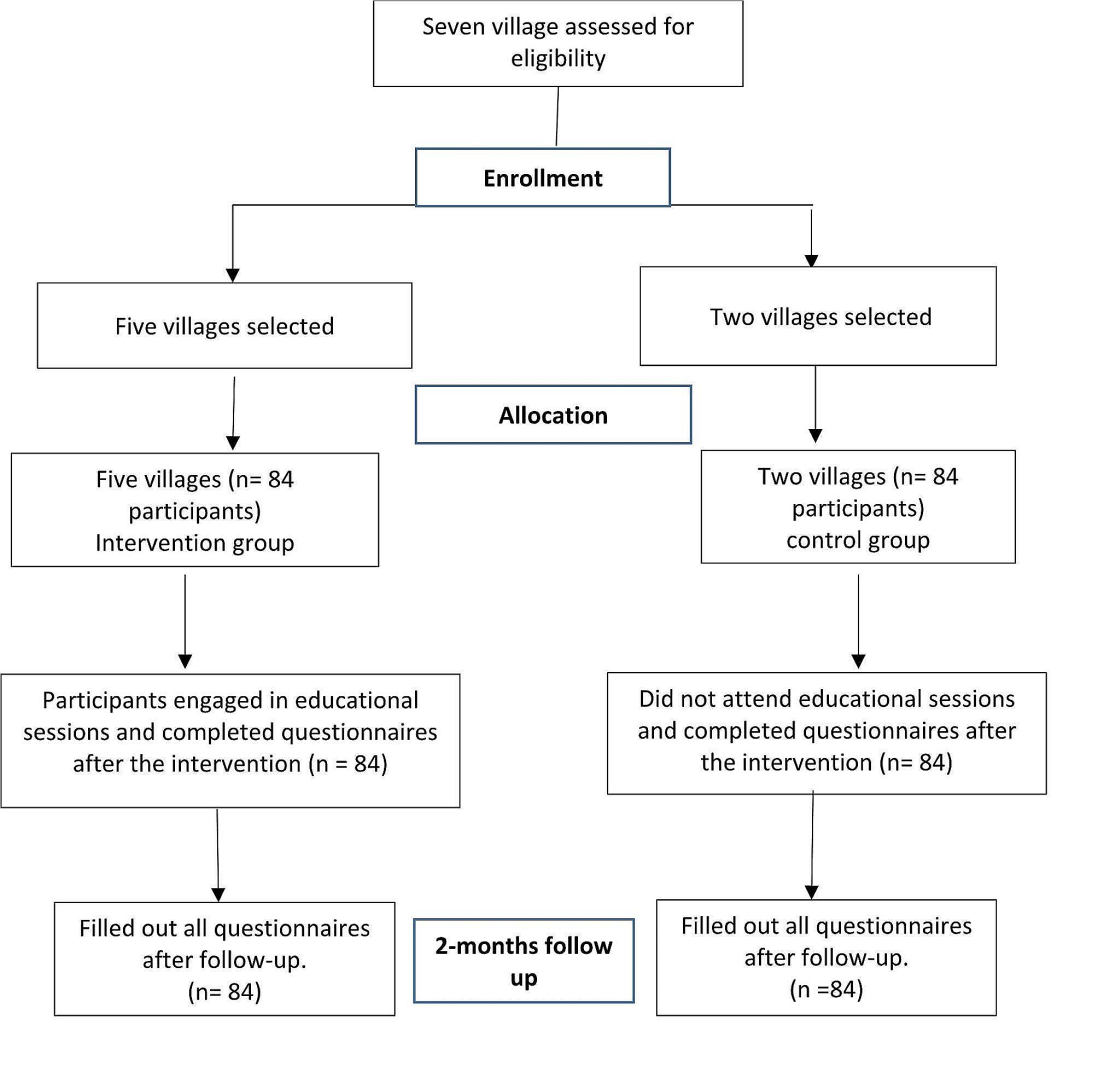
Participant progression through each phase of the program

In this study, endemic villages were considered clusters. Among the endemic villages in north Khorasan, Iran, two villages were randomly allocated for the intervention group and five villages for the control group. Then 84 mothers were assigned randomly to the intervention group and 84 mothers to the control group. No blinding was performed in this study. During the study period, no participants were excluded from the study. Consort checklist is used for Random allocation of villages before individual recruitment. All participants in the study were required to fill out a consent form before participating in the study. The study involved a baseline assessment and a follow-up assessment conducted immediately after the intervention and two months after the intervention. The flow diagram is shown in Fig. 1.

### Intervention

The intervention based on the TIDieR was managed (Format for Intervention explanation and repetition) checklist (Table 1). The community interventions in this study included educational interventions and environmental interventions based on the PPM.

**Table 1:** TIDIeR checklist of the study

Items	Description
**BRIEF NAME**	Theory-based intervention for prompting leishmaniosis prevention behaviors
**WHY** (Rationale of treatment)	To evaluate whether the precede proceed model operating out in the instructional intervention is effective to improve CL prevention behaviors and determined the effectiveness of interventions in the environment to reduce the incidence of leishmaniosis.
**WHAT (**Materials)	The intervention is guided by PPM principles
**WHAT** (Procedures)	This is a quasi-experimental study comprising control and intervention groups. The experimental group received the environmental and educational intervention program. No intervention was carried out in the control group. Eight training sessions were organized, focusing on the key structures of PPM, as detailed in Table 2
**PROVIDER** (Occupation, specialization, background, specialized training)	The training is conducted by expert health educators at the healthcare center
**HOW** (modes of delivery)	Eight educational sessions were conducted face-to-face using the following methods: Lectures, Group discussions, Questions and answers.
**LOCATION (**Infrastructure and relevant features)	Health education and Health promotion service in a healthcare center
**TIMING and QUANTITY** (Number of sessions, duration, intensity or dose)	Each participant engages in a training program include of eight, 90 min sessions, twice a week for one month.
**TAILORING** (Personalization)	Each educational session in the study included standardized components such as question and answer sessions, lectures, educational pamphlets, and focus group discussions, with consistent time and content.
**MODIFICATIONS**	No adjustments were made to the structured intervention during the training
**HOW WELL: planned** (Follow and procedure to preserve it)	All participants received optimal follow-up for the intervention
**HOW WELL: actual**	All participants received the complete planned intervention program without any deviation from the established protocol

Eligible mothers in the educational sections had received eight training sessions (90 min per session) over the course of a month, primarily focusing on key constructs of PPM including knowledge, attitude, reinforcement, and enabling factors. Educational intervention was performend by a specialist health educator at the health care center. In these sessions a health specialist expressed type of leishmaniosis, its risk factors, symptoms, and preventive behaviors toward leishmaniosis. Likewise, review action planning, educational pamphlets, and focus group dissection were used. The details of the educational interventions are listed in Table 2. Mothers in the control group did not receive any educational interventions. We provided training package for control group when the follow-up time and environmental intervention were finished.

The environmental intervention began after the training program and continued for 4 months. The environmental intervention was carried out by the officials of the village council and the health center involving the following activities: 1- The rodent control program was performed 4 times during the study.2- The fight against the disease reservoir was carried out within a radius of 500 m from human places in the village. 3- The hygienic waste collection is done 3 times a week. 4- During the intervention, several time the fertilizer collection strike plan was implemented. 5- Stray dogs were collected and transported to a suitable location. 6- Mosquito repellents and insect repellents were distributed.

**Table 2:** PPM-based educational intervention to improve preventive behavior of CL

Times	Key concepts	Intervention Method	Intervention’s Instruction
**Sessions 1 &2**	Experience and Individual characteristics knowledge	Lecture, PowerPoint, pamphlet, review memories, Review action planning	Definition of the disease and type of leishmaniosis, and risk factors (Living in poor health, existence of construction waste and debris in the living area, Keeping livestock near residential houses), symptoms of the disease, and outbreak season.
**Session 3**	knowledge	Lecture, PowerPoint, pamphlet	Age group at risk, disease process how to infect humans (Sand-fly bites (main way), wound scratching and mechanical transmission by other arthropods (sub way), prevention (install the net on the window, do not leave the house at sunrise and sunset, proper collection and disposal of waste, extermination of rodents in the rural type, lLack of storage and accumulation of animal manure in the residence, use insect repellent ointment)
**Session4**	Attitude	Lecture, Powering, pamphlet	The role and attitude of mothers regarding preventive measures against leishmaniasis, enhancing mothers’ understanding of methods to eliminate carriers and enhance consideration of methods to overcome obstacles
**Session5**	Attitude	Lecture, PowerPoint, pamphlet	Benefits of preventing leishmaniosis, complications of leishmaniosis (scars on the skin and unpleasant appearance, mental health problems, wounds in sensitive areas such as eyelids, ears, lips, and nose; secondary wound infection, drug side effects, costs, and mental health problems), and collective interests of prevention.
**Session6**	Reinforcement factors	Brainstorming group discussion, questions and answers, videos	Approaches to garnering support from family, peers, teachers, and stakeholders, social responsibility of prevention, advise preventive behaviors to others, being able to prevent, and effectiveness of prevention methods
**Session7**	Reinforcement factors	Brainstorming group discussion, questions and answers, videos	The need for timely care and treatment, Lack of home treatment prohibit children from playing in high-risk areas
**Session8**	Enabling factors	Brainstorming group discussion, questions and answers, videos	Waste disposal regulations. Improving prevention skills at home, planning to perform preventive behaviors, avoid preventive behaviors, encourage others to adopt healthy behaviors, express the benefits of preventative behavior, proper garbage collection, and visit. a doctor for follow-up and treatment

### Tools of assessment

The outcome of this research was to progress leishmaniosis prevention behaviors by influencing the mother’s, attitude, knowledge, enabling factors, reinforcing factors, and behavior.

Demographic characteristics included mother’s age, children’s age, mother’s education, father’s education father’s job, mother’s job, and family history of CL.

In this study, we utilized the PPM as a framework to design the questionnaires on Leishmaniosis prevention behavior. We studied the literature to plan the PPM items. Then, all the questionnaires are edited by an expert commission of 10 specialists in infectious disease and health education. They evaluated the importance and relevance of all items to calculate the content validity ratio (CVR) and content validity index (CVI). In this research, the mean CVR for the knowledge, attitude, enabling factors, and reinforce factors and behaviors questionnaires was 0.71,0. 63, 0.075,0. 78, 0.71and CVI for these questionnaires were 0.94, 0.92, 0. 91, 0. 96, 0.87, and 0.90, respectively.

We assessed the readability, clarity, and simplicity of questions based on the pilot study involving 30 mothers. Cronbach’s alpha was calculated to assess reliability. The Cronbach’s alpha values for knowledge, attitude, enabling factors, reinforce factors, and behaviors were 0.70, 0.72, 0.70, 0.75, and 0.53, respectively, that is suggesting a powerful internal constancy of the questionnaire.

Participants in control and intervention groups completed the questionnaires at baseline (before the intervention), immediately after the intervention, and during the 2-month follow-up.

### Knowledge and attitude

On this scale, 18 short items on knowledge of cutaneous leishmaniosis (In what season is rural Leishmaniasis most transmitted? ) with a three-point scale ranged from 0 (no) to 2 (yes), and option I do not know was given a score of 1. (Table 3). The participants’ attitudes were assessed using 12 items. They were asked to rate their agreement with the Attitude Phrase (for example: the use of ointments or creams to repel insects is unpleasant) sing a 5-point scale from 1 (strongly disagree) to 5 (strongly agree) (Table 3).

### Reinforcing factors and enabling factors

We used 8 questions to measure reinforcement factors (My friends encouraged me to use mosquito nets and insect repellent ointments). All questions in these constructs ranged from 0 (no) to 2 (yes), and option I do not know was given a score of 1 (Table 3). Enabling factors constructs include 8 items. (Do you know the local place to buy leishmaniosis prevention devices? ). The items within these constructs were rated on a scale from 0 (no) to 2 (yes), with the option ‘I do not know’ assigned a score of 1.

### Behaviors

Behavioral outcome was assessed using 4 items (Do you use a mosquito net at home? ) with a three-point scale ranging from 0 (no) to 2 (yes), and option I do not know was given a score of 1. Moreover, Table 3 presents the number of questions and the range of scores.

**Table 3:** The number of questions and Score Range

Construct	Number question	range of scores	Balanced from 100
**Knowledge**	18	0–36	0-100
**Attitude**	12	12–60	20–100
**Reinforcing factors**	8	0–16	0-100
**Enabling factors**	8	0–16	0-100
**Behaviors**	4	0–8	0-100

### Statistical analysis

The statistical analysis was performed using SPSS_20_. The Kolmogorov-Smirnov test was utilized to assess differences between two distributions. Additionally, we conducted descriptive analysis (frequency, mean, and standard deviation) and bivariate analyses (paired t-test, Wilcoxon test, and Friedman test) to assess the variability of different variables in both the control and intervention groups. Analysis of variance was used to determine differences among outcomes from baseline to follow-up in intervention and control groups, and *p* < 0.05 was considered as the significance level.

## Results

In this study, the mean age of mothers was 32.72 ± 6.19, children 3.85 ± 4.82 and family size 3.10 ± 1.93. Education level among most fathers and mothers were high school and diploma. Most fathers were self-employed and mother were housewives. The history of CL in the family reported in 91.6% participant.

Details of the participants’ socio-demographic characteristics in control and intervention groups were summarized in Table 4. There was no significant difference in participants’ socio-demographic characteristics (*P* > 0.05) between both groups, except fathers’ jobs, and mother’s and father’s education. The results of the study comparing the mean scores of knowledges, attitude, enabling factors, reinforcing factors, and behavior outcomes in the control and intervention groups at all-time points (baseline, immediately after intervention, and 2-months follow-up) are presented in Table 5. At baseline, the study revealed no significant difference (*P* > 0.05) in the scores of PPM constructs between the control and intervention groups.

**Table 4:** Baseline characteristics of the study population

Variables		Control group (*n* 84)		Intervention group (*n* 84)		total		†*P* Value
		Mean or *N*	SD or %	Mean or *N*	SD or %	Mean or *N*	SD or %	
**Mothers age**		33.13	7.30	32.31	5.08	32.72	6.19	o.399
**Children’s age**		3.75	2.60	3.95	2.22	3.85	4.82	0.312
**Family size**		2.29	1.20	3.92	0.73	3.10	1.93	0.078
**Fathers education**	Illiterate Elementary	37	44	14	16.7	51	30.35	*0.001
	High School & Diploma	41	48.8	50	95.5	91	54.16	
	Associate Degree and Higher	6	7.1	20	23.8	26	15.47	
**Mothers education**	Illiterate& Elementary	30	35.7	8	9.5	38	22.61	*0.001
	High School & Diploma	48	57.1	54	64.3	102	60.71	
	Associate Degree and Higher	22	26.2	6	7.1	28	16.66	
**Fathers job**	employee	4	4.8	19	22.6	23	13.69	*0.001
	Worker	54	26.4	25	29.8	79	47.02	
	Self Employed	26	31.0	40	47.6	76	45.23	
**Mothers job**	household	83	98.8	80	95.2	163	97.02	0.173
	Employee	1	1.2	4	4.8	5	2.97	
**Family history of leishmaniosis**	Yes	74	88.1	80	95.2	154	91.6	0.094
	no	10	1.2	4	4.8	14	8.33	

n, number of eligible participants

* statistically significant at the 0.05 level

†Testing significant change between Intervention and control groups

The mean of knowledge in the intervention group at the baseline was 39.68 ± 15.69. The average knowledge scores of the intervention group immediately after intervention and 2 months of follow-up were 83.93 ± 10.81 and 91.73 ± 9.85, respectively. whereas there was no significant change in the mean knowledge score in the control group throughout the study. The average enabling factors scores of the intervention group in baseline immediately after intervention and 2 months of follow-up were 29.76 ± 19.92, 65.18 ± 17.12 and 75.30 ± 12.79, respectively. This difference was significant (*p* < 0.001).

The mean score of reinforcing factors immediately after intervention) 79.17 ± 22.74(and two months of follow-up (85.12 ± 14.77) showed a significant increase (*p* < 0.001) compared to the baseline. The results showed that the mean of attitude in the intervention group increased from 51.67 ± 11.47 at the baseline to 75.15 ± 7.55 immediately after the intervention and 86.65 ± 6.58 through follow-up(*p* < 0.001). while there was no significant increase in the mean of attitude during the study period in the control group (Table 5).

Our results indicated a significant change in behavioral outcomes among participants in the intervention group (*P* < 0.001) from baseline to follow-up, compared to the control group. while there was no statistically significant variance in the mean behavior scores within the control group throughout the study duration. This suggests that the intervention had a significant impact on modifying behavior outcomes.

The results of analysis of variance in repeated data showed that the attitude score immediately after the intervention in the intervention group increased by an average of 37.47 units, which was a significant difference. Also, two months after the intervention, the average increase was 52.3 units, which was a significant difference (Table 6). The results of analysis of variance in repeated data showed that the performance score immediately after the intervention in the intervention group increased by an average of 20.84 units, which was a significant difference. Also, two months after the intervention, this increase was on average 25.06 units, which was a significant difference. Other results of analysis of variance in repeated data are shown in Table 6.

**Table 5:** Comparison between the mean scores of knowledge, attitude, enabling factors, reinforcing factors, and performance of participants before and after the intervention in the control and intervention groups

Variables PPM construct		Pre intervention		After intervention		Mean difference			2-month follow-up		Mean difference		
		Mean	SD	Mean	SD	Mean	SD	‡p-value	Mean	SD	Mean	SD	§p-value
**Knowledge**	intervention	39.68	15.69	83.93	10.81	44.24	15.29	0.001	91.73	9.85	91.73	9.85	0.001
	control	26.39	13.02	33.27	8.89	6.88	9.15	0.171	28.51	9.32	28.51	9.32	0.194
	different	13.29	2.22	50.66	1.53	37.37	1.94		63.23	1.48	49.93	1.98	
	†p-value	*0.053		0.001		0.001			0.001		0.001		
**Attitude**	intervention	51.67	11.47	75.15	7.55	24.48	12.91	0.001	86.65	6.58	34.98	12.19	0.001
	control	43.65	7.80	37.96	6.04	-5.69	9.19	0.094	33.63	6.16	-10.02	8.82	0.134
	different	8.02	1.51	38.19	1.05	30.18	1.73		53.02	0.98	45.00	1.64	
	†p-value	0.052		0.001		0.001			0.001		0.001		
**Enabling factors**	intervention	29.76	19.92	65.18	17.12	35.42	20.68	0.001	75.30	12.79	45.54	19.99	0.001
	control	35.87	17.26	31.10	13.23	-6.77	17.95	0.001	36.09	16.22	-1.79	17.90	0.001
	different	-8.11	2.88	34.08	2.36	42.19	2.98		39.21	2.25	47.32	2.93	
	†p-value	0.06		0.001		0.001			0.001		0.001		
**Reinforcing factors**	intervention	27.5	21.56	79.17	22.74	51.67	0.24	0.001	85.12	14.77	57.62	26.00	0.001
	control	22.50	21.82	22.74	12.16	0.24	19.32		29.40	18.25	6.90	14.14	0.05
	different	5.00	3.27	56.43	2.34	51.42	3.79		55.71	2.56	50.71	3.23	
	†p-value	0.054		0.001		0.001			0.001		0.001		
**Behavior**	intervention	51.93	13.96	85.57	9.80	33.63	16.72	0.001	92.26	7.86	40.33	15.01	0.001
	control	45.39	15.34	34.90	14.40	10.49	13.69	0.006	44.12	15.26	-1.26	11.75	0.013
	different	6.55	12.26	50.66	1. 90	44.12	3.36		48.14	1.87	41.59	2.08	
	†p-value	0.053		0.001		0.001			0.001		0.001		

†Assessing significant differences between control and intervention groups

‡ Examining significant changes in the intervention and control group before and after the intervention

§ Assessing significant changes in the intervention and control group before and at the 2-month follow-up

**Table 6:** Analysis of repetitive data on knowledge, attitude, enabling factors, reinforcing factors, behavior in the experimental and control groups before and 2 months after the intervention

		knowledge			attitude			Enabling factors			Reinforcing factors			behavior		
		value	SE	P-value	value	SE	P-value	value	SE	P-value	value	SE	P-value	value	SE	P-value
**After intervention**	**Pre intervention**	0.32	0.05	< 0.001	0.09	0.05	0.095	0.30	0.06	< 0.001	0.29	0.06	< 0.001	0.31	0.061	< 0.001
	Intervention group	44.71	1.45	< 0.001	37.47	1.13	0.001	36.49	2.26	< 0.001	36.49	2.26	< 0.001	48.64	1.82	< 0.001
	Control group	0*	0		0*	0		0*			0*	0		0*	0	
**2-month follow-up**	**Pre intervention**	0.30	0.05	< 0.001	0.12	0.05	< 0.001	0.30	0.06	< 0.001	0.29	0.06	< 0.001	0.42	0.06	< 0.001
	Intervention group	57.88	1.45	< 0.001	52.03	1.05	< 0.001	41.55	2.15	< 0.001	41.55	2.26	< 0.001	45.39	1.66	< 0.001
	Control group	0*			0*			0*			0*			0*		

*Control group is Base variable

## Discussion

We conducted this study to assess the effectiveness of an educational intervention based on PPM constructs in promoting CL prevention behaviors among mothers with children under 10 years of age. Our findings indicated no significant difference in scores of the PPM’s constructs between the control and intervention groups at baseline. This suggests that confounding factors had a minimal impact on the research outcomes. The results of this study showed that education and environmental intervention based on PPM significantly promoting mothers’ prevention behaviors toward CL by changing their attitudes, knowledge, and modifying enabling and reinforcing factors. Other studies, such as the study by Jeihooni and colleagues, also showed that the application of the PPM can be effective in improving the behavior of the CL [10, 26].

Before the intervention, participants had less knowledge about the prevention behaviors (39.68 out of 100). People in rural areas have less access to information resources, and many of their behaviors are due to insufficient knowledge. Several factors help mother to take preventive measures such as knowing the outbreak season [20, 33], the cause of the disease, the symptoms of the disease [10, 17], and the route of transmission [21, 33]. In many studies, lack of knowledge about clinical presentation, disease transmission, prevention, and treatment [29], disease symptoms, disease vector [34], and control measures, outbreak season has been introduced as a factor to increase the prevalence of CL [7, 35].

Participants’ attitudes were low before the intervention (51.67 out of 100). Lack of sufficient knowledge about the CL and the beliefs such as using local methods for treatment, getting help from local therapists, spontaneous treatment were more common in rural areas [9, 22]. In other studies, the belief in the use of traditional medicine [36] in the treatment of CL and the rejection of medical treatment was one of the common beliefs that delayed preventive behavior [37].

Before the intervention, respondents reported low enabling factors (29.76 out of 100). Rural areas are usually a long distance from the city and shopping malls, so there was no access to proper equipment and facilities to control the disease [11]. In Tamiru’s study, factors such as access to medical facilities, distance led to the choice of traditional treatment for the disease [2, 38]. Before to the intervention, respondents identified weak reinforcing factor (27.5 out of 100). In rural areas, the number of people who knew, used and encouraged the use of disease prevention tools was small. It is also difficult to change the customs of the society in rural communities. Usually, people with certain influential characteristics such as dean have the ability to promote and encourage new behavior [2].

Participants had poor behavior before the intervention (51.93 out of 100). In addition to keeping livestock in the house, they did not use mosquito nets and repellent ointments. In similar studies, the lack of preventive behaviors such as the use of netting for doors and windows, ad mosquito nets were identified as important factors in the spread of the disease [7, 39]. The use of traditional therapies was also one of the inappropriate behaviors that prolonged the treatment period [22, 40]. Although, this information seems simple, but if taught in a theoretical framework lead to a significant impact on the quality of life, especially in rural areas.

The educational program was done for a month. In the one-month research project, participants have the opportunity to familiarize themselves with different aspects of the disease and think about the usefulness of prevention methods and decide to change [11, 22]. Consider whether they need to correct their beliefs towards preventive behaviors. To increase the effectiveness of the intervention various strategies have also been used to attract participants, such as movie screenings, which helps to improve attitude [2, 41]. We used different strategies to improve behavior that is useful for behavior change, advocacy, practical actions of health care providers, providing the necessary equipment, holding training classes, distributing pamphlets, and educational booklets. The results are consistent with other studies [9, 21]. In addition, we conducted activities such as distributing mosquito nets, collecting garbage, controlling rodents, and collecting stray dogs. They seem to have been effective in increasing the effectiveness of educational interventions [42].

### Implication and policy recommendations

Evaluating the effectiveness of the theoretical model in different educational interventions would be effective for better understanding the potential determinate or mediator to control and prevent burden and actual incidence of CL. Likewise, we investigated how, where and when determinants eliminate children’s exposure to CL and change mothers’ and children’s preventive behaviors and outcomes to follow positive behaviors. Performing environmental interventions was one of the main strengths of the study, which is neglected in most studies, but in this study, it was used in addition to educational interventions. In this study, environmental interventions based on PPM are part of an intervention to evaluate and modify the extent and origin of CL in the child and mother populations. Thus, it is essential for health manager and policy maker to more attention to theory-based training strategies and environmental intervention to improve individual’s preventive behaviors toward CL and health outcomes in the medical care context.

### Strengths and limitations

One strength of this study lies in its inclusion of environmental interventions alongside educational interventions. A unique aspect of the present study was the maintenance of all positive intervention effects at the 2-month follow-up. In this study, we faced some limitations. First, the self-report questionnaire was used, possibly leading to biases in final outcomes. Second, mediator questions were used to design questionnaires that may not reflect all aspects of PPM’s constructs in a natural setting. However, reliability was more than 0.9 for questionnaires. Third, we could not assess the long-term behavior of the participants (6 months and 12 months), especially, after the end of the environmental intervention. Fourth, we could not select more sample sizes from the mothers, but the sample size was estimated not to distort the results.

## Conclusion

Educational and environmental intervention for mothers not only increased the preventative behavior of leishmaniosis in mothers but also was efficacious in reducing the incidence of disease in children. Our findings showed that educational intervention based on PPM is feasible and acceptable to modify preventive behaviors of CL in children by increasing a mother’s knowledge and attitude as well as changing enabling and reinforcing factors. Therefore, further intervention research-based PPM is needed to promote a positive behavior change and identify significant mediators.

## Data Availability

The data sets utilized and/or analyzed during the present study ere available from the corresponding author upon reasonable request.

## Abbreviations

PPM: Precede proceed Model
CL: Cutaneous leishmaniosis
WHO: World Health Organization
CVI: Content Validity Index
CVR: Content Validity Ratio
